# Behavioral representations within the endogenous dual attentional pathways during audiovisual integration processing

**DOI:** 10.3389/fnins.2025.1536688

**Published:** 2025-02-11

**Authors:** Zhongtian Guan, Mingli Yan, Miao He, Yubo Liu, Zhixi Zhang, Chunlin Li

**Affiliations:** 1 School of Biomedical Engineering, Capital Medical University, Beijing, China; 2 Institute of Large-Scale Scientific Facility, Beihang University, Hangzhou, Zhejiang, China; 3 Aerospace Information Research Institute, Chinese Academy of Sciences, Beijing, China

**Keywords:** fMRI, endogenous attention pathway, audiovisual integration, Posner cueing paradigm, behavioral representations

## Abstract

**Introduction:**

Audiovisual integration processes are modulated by top-down attention and may involve different neural mechanisms under spatial, temporal, and neutral properties, which are externally manifested by subjects adopting different cognitive strategies. Composite behavioral indicators are able to assess the balance between speed and accuracy when subjects perform a task, thus further revealing behavioral representations of cognitive tasks. However, the cognitive strategies and neural mechanisms involved in audiovisual integration under endogenous attentional modulation are unclear, and in particular, the functional relationship between the dorsal and ventral pathways still needs to be thoroughly investigated.

**Methods:**

We design only auditory (A), only visual (V), and audiovisual attention (VA) tasks based on the classical Posner paradigm with spatial, temporal and neutral cues for behavioral indicators, brain activation, and their correlations.

**Results:**

Our results showed significant differences in behavioral performance between tasks, with weaker performance on the dual-channel task than on the single-channel task. The brain showed consistent activation in the frontal eye field, inferior parietal lobule, supplementary motor area, superior temporal gyrus, middle occipital gyrus and cuneus. The dorsal pathway is mainly associated with spatial processing and executive control, while the ventral pathway is involved in object recognition and semantic processing. In correlation analyses, proportions of correct responses (PC) showed a wider range of results. Spatio-temporal attention mobilized more cognitive resources than neutral attention in the audiovisual task and influenced execution strategies. Inverse efficiency score (IES) revealed endogenous attentional modulation of energy expenditure in the dual-channel task, whereas rate-correct score (RCS) revealed inter-task differences in correct response efficiency. Linear integrated speed–accuracy score (LISAS) and balanced integration score (BIS) showed different speed-accuracy balance in our task.

**Conclusion:**

Our findings emphasize the synergy of functions and the dynamic synergy of cognitive processes in dorsal and ventral attentional pathways, which contribute to the management of cognitive flexibility and efficiency.

## 1 Introduction

Visual and auditory information are the two main sources of external information for humans, accounting for about 90 percent of the total (Treichler, 1967). The phenomenon of audio-visual integration exists everywhere in people’s lives, such as the integration of sound and subtitles when watching TV, the integration of speech and oral information when communicating with others, and the effective integration of information from the visual and auditory channels into a unified and coherent perceptual (Tang et al., 2016; Parker and Robinson, 2018). Audiovisual integration is able to integrate ambiguous information to make better inferences about external connections (Ernst and Bülthoff, 2004; Noppeney, 2021) and thus plays an important role in the human brain’s understanding of external information.

Audiovisual integration is influenced by many factors, including spatial (Nidiffer et al., 2016; Spence and Frings, 2020), temporal (Stevenson and Wallace, 2013), and attentional (Donohue et al., 2015; Mole, 2020) factors. Attention has top-down or bottom-up control over multisensory integration (Meyer et al., 2018), and endogenous, goal-driven attention, mediated by the dorsal attention network, is particularly effective in enhancing audiovisual integration under attended conditions (Talsma et al., 2007; Talsma et al., 2010). Whereas exogenous attention, often controlled by the ventral attention network, tends to override endogenous cues, resulting in reflexive spatial localization (Tang et al., 2016). These attentional processes guide the orientation of attention, with studies showing that the frontoparietal network, including both dorsal (for spatial attention) and ventral (for stimulus-driven attention) pathways attention (Landry et al., 2024), proposed by Posner (1980) in 1980, demonstrating that when cues produce short intervals between the cue and the target (< 250 ms), prior exogenous cues facilitated the subject’s response to the next target, but may also produce Inhibition of return (Van der Stoep et al., 2017). An fMRI study (Yan et al., 2015) measured cortical areas activated in the visual and auditory domains in a cue-target spatial attention paradigm. The results suggest that interactions between multisensory inputs can lead to enhancement or inhibition of cortical responses with top-down spatial attention. Thus audiovisual integration and attention are both important mechanisms, capable of recognizing the external world by enhancing sensory perception (Spence, 2010), and their interaction is particularly important (Talsma et al., 2010; Tang et al., 2016).

In the field of experimental psychology, it is common to use mean response time (RT) and proportions of correct responses (PC) to ideally quantify subject behavioral performance, however, it does not take into account the subject’s tendency to answer the questions, whether their strategy is to be more precise or faster at completing the task, which brings us to the speed-accuracy trade-off (SAT) (Luce, 1991; Heitz, 2014). SAT suggests that intra-or inter-subject trade-offs are often unpredictable (Gueugneau et al., 2017), For example, some researchers have focused on the ‘posterior response delay’ that occurs when subjects make a mistake (Thura et al., 2017). Thus, RT or PC are often very vague concepts, and by integrating them we can further focus on behavioral changes in one direction or another (Liesefeld et al., 2015). Inverse Efficiency Score (IES), Rate-Correct Score (RCS), Linear Integrated Speed-Accuracy Score (LISAS) and Balanced Integration Score (BIS) are important composite behavioral indicators for evaluating behavior in the field of psychology (Liesefeld and Janczyk, 2019).

Functional connectivity has been shown to correlate with transient mental states (Quinn et al., 2018), and the extent of its influence on task performance can also be established (Sadaghiani et al., 2015). However, this approach only reveals fixed intrinsic connectivity and behavioral properties at rest-state. In contrast, task-state fMRI allows for a more accurate characterization of behavioral states in a given cognitive direction and establishes a more direct link to behavioral contributions through local neural activity. Despite this, it remains unclear how different types of attention affect the behavioral strategies for audiovisual integration, particularly the shifting focus between accuracy and speed. Furthermore, how brain regions within the dorsal and ventral attention pathways fine-tune their functions and correlate with behavioral performance still requires further exploration.

Therefore, our study aims to establish the link between brain activity and behavioral representations of audiovisual integration under endogenous attentional conditioning. By combining basic and composite behavioral indicators, the study applies task-state fMRI to design three tasks—auditory, visual, and audiovisual—under three types of endogenous attention: spatial, temporal, and neutral, based on the classical Posner experimental paradigm. Behavioral and fMRI data were analyzed to explore differences in behavioral indicators and activated brain regions across the A, V, and VA tasks under spatial, temporal, and neutral attention conditions. The goal is to uncover the functional characteristics of the dorsal and ventral attention pathways in audiovisual integration and to analyze the key brain regions associated with behavioral indicators in the attention network, clarifying their regulatory mechanisms.

## 2 Materials and methods

### 2.1 Participants

52 young people aged 18–27 years with normal vision, hearing and cognitive abilities were recruited for this study, with the following inclusion criteria: right-handed, normal vision or corrected vision, normal hearing, and no history of brain injury; no history or diagnosis of psychiatric disorders; no use of psychotropic medications; and no contraindications to MRI (e.g., claustrophobia and metal implants). Considering the completeness of data collection, 49 of the subjects (mean age 22.7 years, 20 males, 29 females) were included in the analysis, including complete behavioral data and task-state fMRI data, which were collected from the radiology department of Beijing Youan Hospital affiliated with Capital Medical University. The study was approved by the Ethics Committee of Capital Medical University, and each subject was informed of the experimental details and signed a written informed consent form prior to the study, and received a certain amount of transport compensation.

### 2.2 Experimental paradigm

The cue and target stimulus were designed according to the classical Posner experimental paradigm (Posner et al., 1980), where a cue stimulus and a target stimulus are included in a trial. The visual display comprised a central cueing stimulus (1° eccentricity) and the bilateral white peripheral boxes (7° eccentricity) in which the visual target stimulus (“x”) appeared (Coull and Nobre, 1998). The additional auditory target stimulus was white noise from 20 to 20,000 Hz. Auditory and visual signals were generated in the experiment via Presentation 22.0.^1^ Spatial cue (S), temporal cue (T), and neutral cue (N) were included in the cued stimuli for this experiment (Li et al., 2012). Specifically, the spatial cue was designed as the highlighted half of the diamond in the middle of the screen, pointing to the left or right, to prompt participants to the possible location of the next target stimulus presented. The temporal cue was the small and large highlighted concentric circle in the middle of the screen, to call attention to the length of the gap between the cue and the target stimulus. The small circle stood for the interval of 300 ms, while the large circle stood for the interval of 1,500 ms. To remind participants to pay attention, all lines in the central screen were designed to be highlighted as a neutral cue.

**Figure 1:**
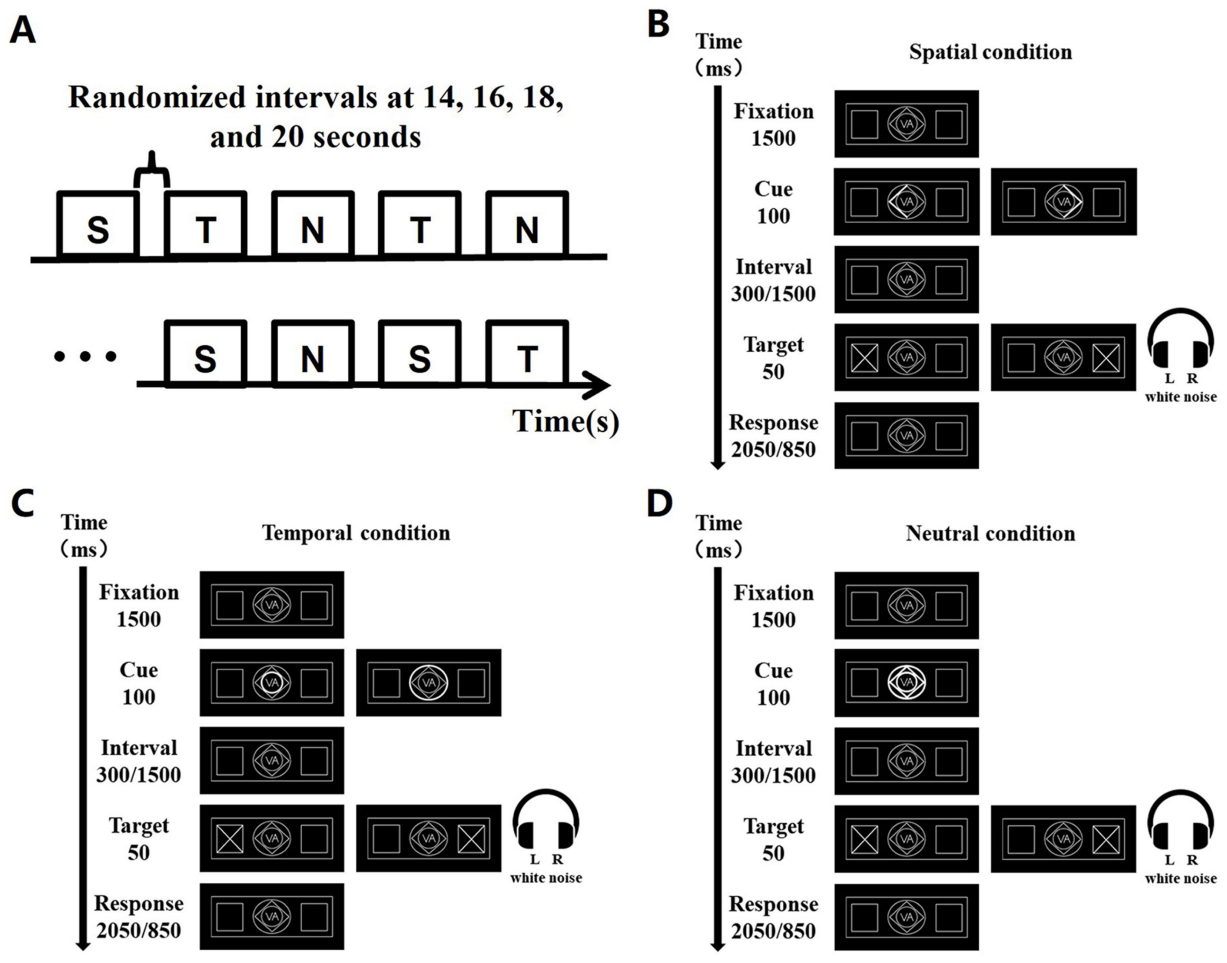
Task experimentation paradigm, exemplified by the VA task. **(A)** 9 blocks were acquired for each task, and the order of the blocks was S, T, N, T, N, S, N, S, T. S denotes spatial cues, T denotes temporal cues, and N denotes neutral cues. A random baseline gap of 14, 16, 18, and 20 s exists between every two blocks. **(B)** Presentation of each trial over time under spatial cues. **(C)** Presentation of each trial over time under temporal cues. **(D)** Presentation of each trial over time under neutral cues.

The paradigm design is shown in Figure 1, with subgraph A showing that each task consists of 9 blocks, each of which in turn consists of 10 trials. There were peaceful black screen intervals between blocks in a random interval of 14 s, 16 s, 18 s, and 20s. All trials in each block had the same type of cue stimuli. Three groups of blocks are arranged in a cyclic sequence three times, and in order to eliminate the effect of the order of S, T and N: S, T, N, T, N, S, N, S, T. The temporal design of each trial is shown in Figures 1B–D. Each trial was 4 s and was arranged by fixation point background, cue stimulus, background, target stimulus, and final background. The duration of the fixation point background was 1,500 ms, the cue duration was 100 ms, the interval between the cue and the target was either 300 ms or 1,500 ms, the target stimulus presentation was 50 ms, and the final background was either 2050 ms or 850 ms as a supplement to the total 4-s duration of the trial.

The A task gave signals to the auditory channel only; the V task gave signals to the visual channel only; and the VA task gave signals to both the visual and auditory channels. The experiment required subjects to draw attention to a cue stimulus when it appeared and subsequently make a button response when the target stimulus appeared, i.e., in the A task, subjects were required to press the button that indicated the side direction of sound appearance. In the V task, subjects were required to press the button that indicated the side direction of the appearance of the diagonal cross line. In the VA task, subjects were required to press the button that indicated the simultaneous appearance of the side direction response button for both sound and visual signals.

Participants were informed of all relevant details before the experiment and completed a pre-experiment session to familiarize themselves with the task paradigm. During the main experiment, they lay in the MRI scanner equipped with MRI-compatible audiovisual equipment, including earphones connected via audio cables to transmit auditory signals and eyepiece screens with interchangeable lenses connected to video cables to deliver visual stimuli. Participants held a custom response device in their right hand, with the index finger assigned to the left button and the ring finger to the right button, connected to an external computer outside the magnetic field. Synchronization was achieved via a trigger mechanism. Prior to each task, participant comfort was assessed and adjusted as necessary using MRI-compatible audiovisual communication equipment.

### 2.3 MRI data collection

The fMRI data were acquired using a 3.0 T MRI scanner (GE SIGNA Pioneer) with a 32-channel head coil. Functional images were collected with the following scanning parameters: repetition time (TR) of 2,000 ms, echo time (TE) of 30 ms, flip angle (FA) of 90°, slice thickness of 3.5 mm with no inter-slice gap, field of view (FOV) of 24 cm × 24 cm, matrix size of 64 × 64, across 36 slices. Additionally, T1-weighted high-resolution structural images covering the entire brain were obtained with a TR of 7.032 ms, TE of 3.016 ms, FA of 12°, number of excitations (NEX) of 1.0, FOV of 24 cm × 24 cm, matrix size of 256 × 256, slice thickness of 1.0 mm without inter-slice gap, yielding 192 slices. For participants with larger head sizes, adjustments were made as necessary.

### 2.4 The composite behavioral indicators

In the field of experimental psychology, Mean RT and PC are commonly used by researchers to quantify subject task performance. However, with the change of task mode, a single indicator cannot fully reflect the individual’s behavioral characteristics and cognitive state, so the composite performance indicators were introduced to comprehensively assess the subjects’ behavioral performance:

1. Inverse efficiency score (IES)

The IES (Bruyer and Brysbaert, 2011) is one of the most commonly used metrics to unify speed and accuracy and is defined as the average reaction time for a correct response divided by the percentage correct in Equation (1):

$$\mathrm{IE}S_{i,j}=\frac{\bar{RT_{i,j}}}{PC_{i,j}}$$ (1)

where *i* denotes the subject and *j* denotes the task, $\bar{RT_{i,j}}$ indicates the mean RT for the *i* subject to respond correctly to the *j* task or condition; *PC_i,j_* denotes the correctness of the *i* subject under the *j* task. The average energy expended by subjects during the trial was characterized by the IES; the IES gradually increased with increasing cognitive difficulty.

2. Rate-correct score (RCS)

The RCS (Woltz and Was, 2006) is defined as the number of correct responses per unit time and Equation (2) is:

$$\mathrm{RC}S_{i,j}=\frac{NC_{i,j}}{\sum_{k=1}^{n_{i,j}}RT_{i,j,k}}$$ (2)

where *i* denotes the subject and *j* denotes the task, *NC_i,j_* denotes the number of times the *i* subject responded correctly in the *j* task or condition, and the denominator is the sum of the RT of all trials of subject *i* in task *j*, the sum of the RT of all the *n_i,j_* trials of the *i* subject in the *j* task or condition. The RCS characterizes the production of correct response efficiency; the RCS decreases as cognitive difficulty increases.

3. Linear Integrated Speed-Accuracy Score (LISAS).

The LISAS (Vandierendonck, 2017) is based on a general linear model that linearly combines the mean RT with the correctness rate, which is formulated in Equation (3) as follows:

$$\text{LISA}S_{i,j}=\bar{RT_{i,j}}+\frac{S_{RT_{i,j}}}{S_{PE_{i,j}}}PE_{i,j}$$ (3)

where *i* denotes the subject and *j* denotes the task, $\bar{RT_{i,j}}$ denotes the mean RT of the *i* subject responding correctly in the *j* task or condition; *PE_i,j_* denotes the proportion error (PE) of subject *i* in condition *j*, with a value equal to 1*-PC*. $S_{RT_{i,j}}$ and $S_{PE_{i,j}}$ are the sample standard deviations for all trials of subject *i* under task *j.* The denominator is *n*, not the biased estimate *n-*1, calculated as $S_{\mathrm{PE}}=\sqrt{\mathrm{PE}(1-\mathrm{PE})}$ . Although both IES and RCS combine RT and PC into one metric and are easy to interpret (i.e., average energy consumed and efficiency of correct response), both are nonlinear, whereas LISAS provides a linear metric. And the larger the LISAS, the weaker the characterization of task switching capability.

4. Balanced integration score (BIS)

The BIS (Vandierendonck, 2018) is defined as the standardized correct rate minus the standardized mean RT, with the following Equation (4):

$$\begin{array}{l} \mathrm{BI}S_{i,j}=z_{PC_{i,j}}-z_{\bar{RT_{i,j}}}\\ z_{x_{i,j}}=\frac{x_{i,j}-\bar{x}}{S_{x}} \end{array}$$ (4)

where *i* denotes the subject and *j* denotes the task, where *PC_i, j_* denotes the rate of correctness of the *i* subject under the *j* task, and $\bar{RT_{i,j}}$ denotes the mean RT for the *i* subject to respond correctly under the *j* task or condition. $z_{x_{i,j}}$ denotes the result after standardization of *x_i,j_*. Performance strengths and weaknesses, or the difficulty of conditions are characterized by BIS. As cognitive difficulty increases, BIS decreases (Posner et al., 1980).

As subjects completed the three tasks A, V, and VA, the computer automatically recorded the reaction time and correct situation for each trial. Based on the reaction time and correctness, the composite behavioral indicators under the three tasks of A, V and VA under S, T and N cues were calculated, respectively.

### 2.5 Data analysis

For behavioral data, two-way repeated measures ANOVA with three levels of each factor was used to compare the differences between the three tasks VA, V, and A under the three attentional cues at the *p* < 0.05 statistical level. The Geisser–Greenhouse method was applied for the correction to account for violations of sphericity, and Tukey’s test was used for post-hoc comparisons to control for multiple comparisons. This statistical approach allowed for the examination of the effects of task type and attentional cues on behavioral performance. For task-state fMRI data, SPM12^2^ based on the MATLAB 2018a platform was used to perform preprocessing. Specifically, removing the first 6 time points to eliminate the effects of artefacts arising from magnetic field instability; temporal layer correction to remove the effects of temporal aberrations on the images; performing head movement correction to remove subjects with head motion translations greater than 3 mm and rotations greater than 3°; spatial normalization to resample to 3 × 3 × 3 mm3; and a 6 × 6 × 6 mm3 smoothing operation. With our sufficiently large number of subjects (49 available), low inter-subject variability, and high sample robustness, a full width at half maximum (FWHM) of 6 mm was effective in balancing spatial smoothing and signal fidelity and retaining sufficiently detailed information. In addition, the experimental design required observation of smaller brain regions contained in the bilateral attentional pathway, so a relatively small FWHM could reduce the loss of excessive smoothing (Mikl et al., 2008). The SPM toolkit was used to calculate the contrast of the two factors. Differences in brain activation for the three tasks A, V, and VA under the stimuli of S, T, and N were obtained separately. AlphaSim correction was used for multiple comparisons to achieve the correction thresholds correspond to a corrected *p* < 0.05 determined by the Monte Carlo simulation (cluster connectivity criterion rmm = 5) by a combination of a voxel-wise level of *p* < 0.01, with a minimum clusters >38 voxels with the program AlphaSim in AFNI (Ward, 2000). To further investigate the relationship between brain region activation levels and behavioral representations, Person correlation analyses of activation *β*-values for the three tasks VA, V, and A under each of the three attentional cues were performed with behavioral metrics using RESTplus (v1.30^3^) which corrected also by AlphaSim in AFNI. To visualize the statistical results, Xjview10.0 was used for brain region labelling.

## 3 Results

### 3.1 Behavioral results

**Figure 2:**
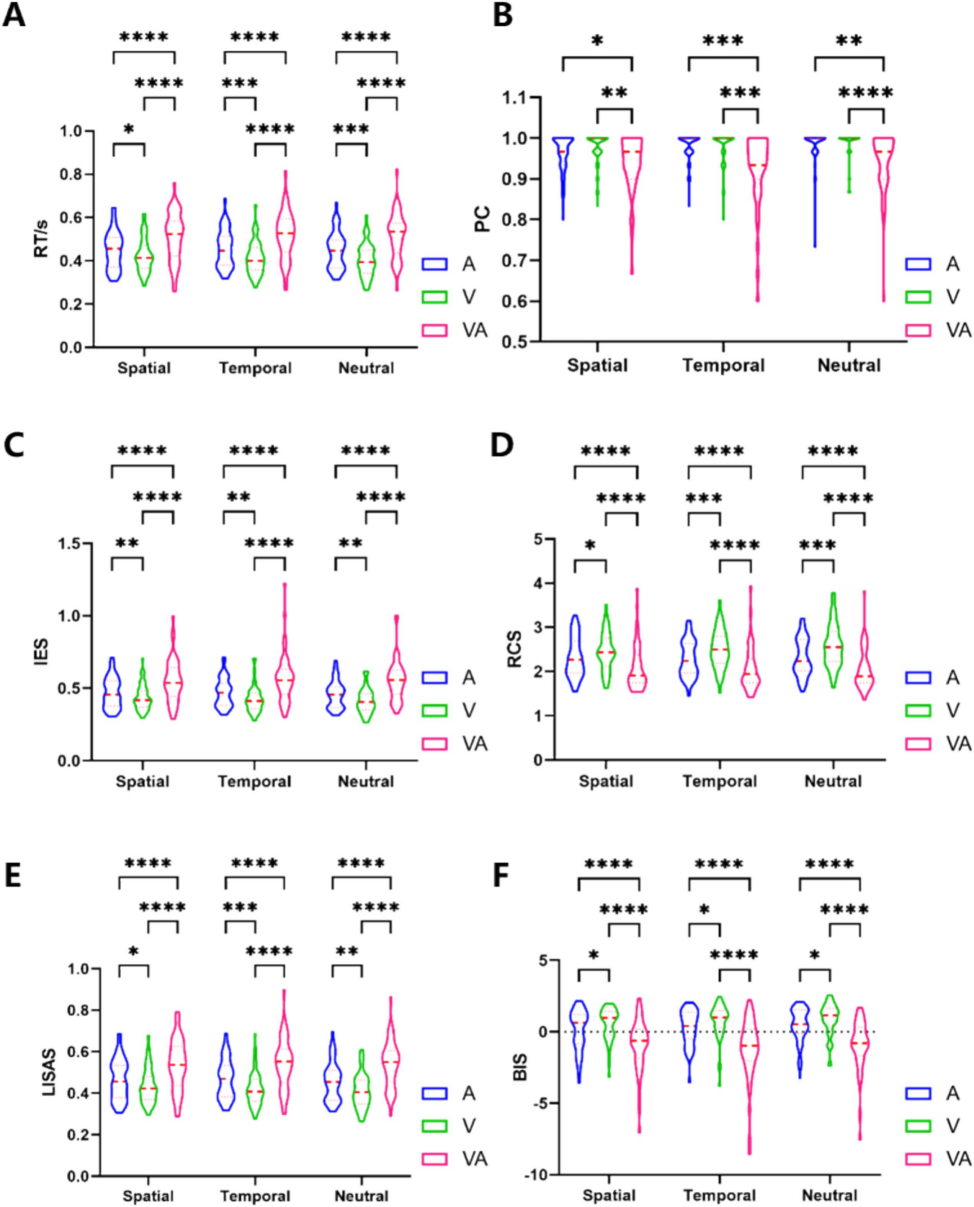
Results for 6 behavioral indicators. **(A)** RT behavioral results. **(B)** PC behavioral results. **(C)** IES behavioral results. **(D)** RCS behavioral results. **(E)** LISAS behavioral results. **(F)** BIS behavioral results. *Denotes *p* < 0.05, ** denotes *p* < 0.01, ***denotes *p* < 0.001, ****denotes *p* < 0.0001.

Calculated values for 6 indicators RT, PC, IES, RCS, LISAS and BIS are presented in Figure 2 and Table 1 as mean ± standard deviation according to the three tasks A, V and VA under S, T and N cues, respectively. A within-subjects group statistical analysis was performed using GraphPad prism 9.4.1 (Geisser–Greenhouse method for the correction, Tukey test, p < 0.05). In order to explore differences in behavioral performance between the VA and A, and VA and V tasks, the study was described separately in terms of spatial, temporal and neutral cues.

**Table 1:** Means and standard deviations of behavioral indicators.

Behavioral indicators	S			T			N		
	A	V	VA	A	V	VA	A	V	VA
RT	0.45 ± 0.087	0.42 ± 0.079	0.51 ± 0.111	0.46 ± 0.087	0.41 ± 0.076	0.52 ± 0.110	0.45 ± 0.086	0.40 ± 0.076	0.52 ± 0.106
PC	0.96 ± 0.049	0.97 ± 0.041	0.94 ± 0.069	0.98 ± 0.036	0.98 ± 0.042	0.93 ± 0.087	0.98 ± 0.044	0.98 ± 0.038	0.94 ± 0.074
IES	0.47 ± 0.104	0.44 ± 0.088	0.55 ± 0.147	0.47 ± 0.098	0.42 ± 0.090	0.57 ± 0.169	0.46 ± 0.094	0.41 ± 0.085	0.55 ± 0.140
RCS	2.32 ± 0.449	2.45 ± 0.435	2.09 ± 0.518	2.28 ± 0.413	2.51 ± 0.436	2.06 ± 0.497	2.32 ± 0.429	2.58 ± 0.484	2.06 ± 0.463
LISAS	0.47 ± 0.097	0.44 ± 0.084	0.54 ± 0.122	0.47 ± 0.094	0.42 ± 0.082	0.54 ± 0.123	0.46 ± 0.093	0.41 ± 0.081	0.54 ± 0.113
BIS	0.12 ± 1.419	0.55 ± 1.123	−0.94 ± 1.836	0.29 ± 1.226	0.75 ± 1.201	−1.20 ± 2.142	0.45 ± 1.224	0.89 ± 1.117	−0.92 ± 1.757

### 3.2 Differences in brain region activation

**Figure 3:**
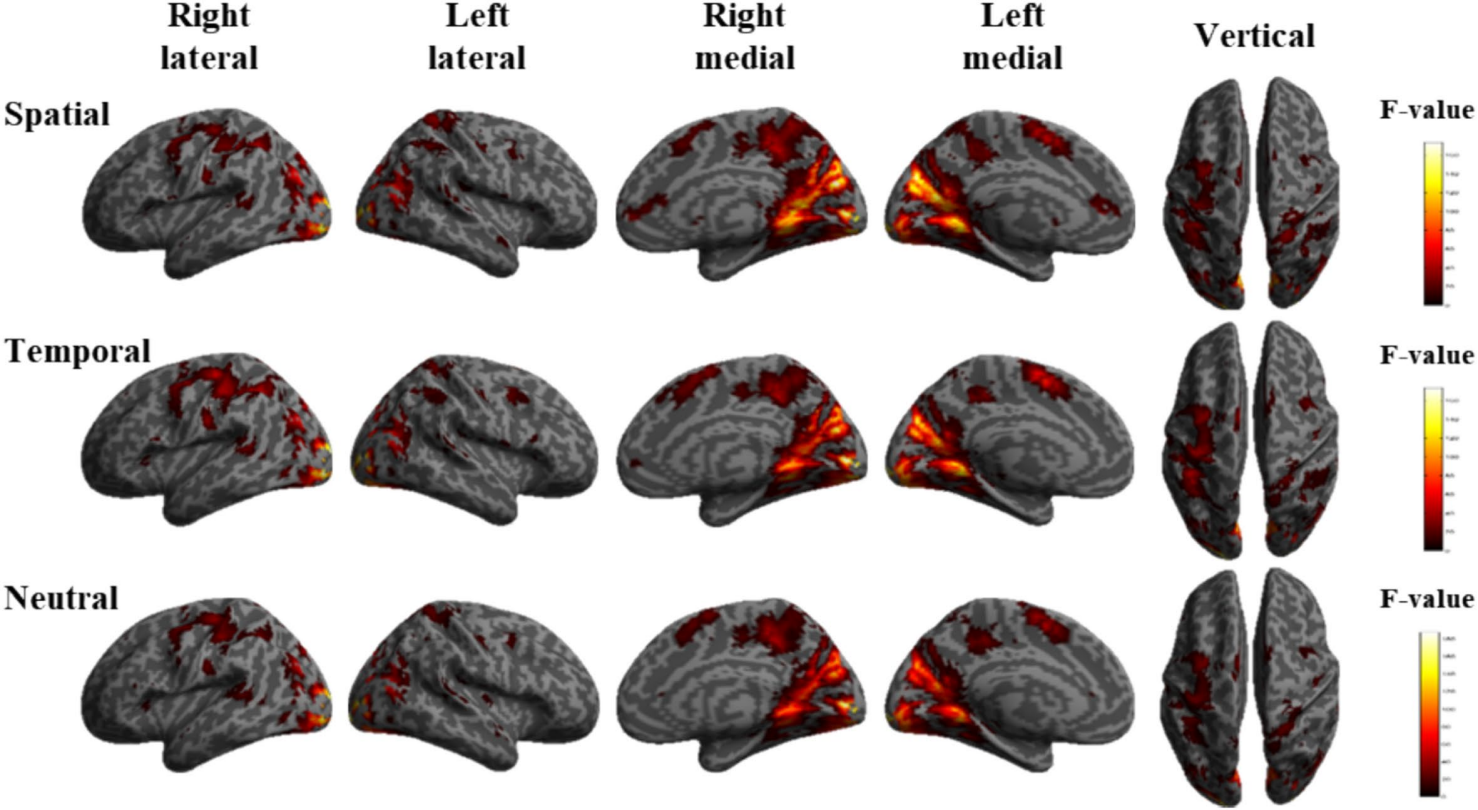
Brain activation maps of the simple effects of audiovisual factors under spatial, temporal and neutral cues.

To compare the brain activation differences under the three tasks A, V and VA, the simple effects of audiovisual factors were analyzed under the three cues S, T and N, respectively. The locations of the activation difference brain regions are shown in Figure 3. Under spatial cues, the activated brain areas were mainly located in the Frontal Eye Field (FEF), Inferior Parietal Lobe (IPL), Supplementary motor area (SMA), Superior Temporal Gyrus (STG), Middle Occipital Gyrus (MOG), Cuneus (CUN). Activated brain regions were largely consistent with spatial cues under temporal and neutral cues.

### 3.3 Correlation analysis of task activation with behavioral indicators

**Figure 4:**
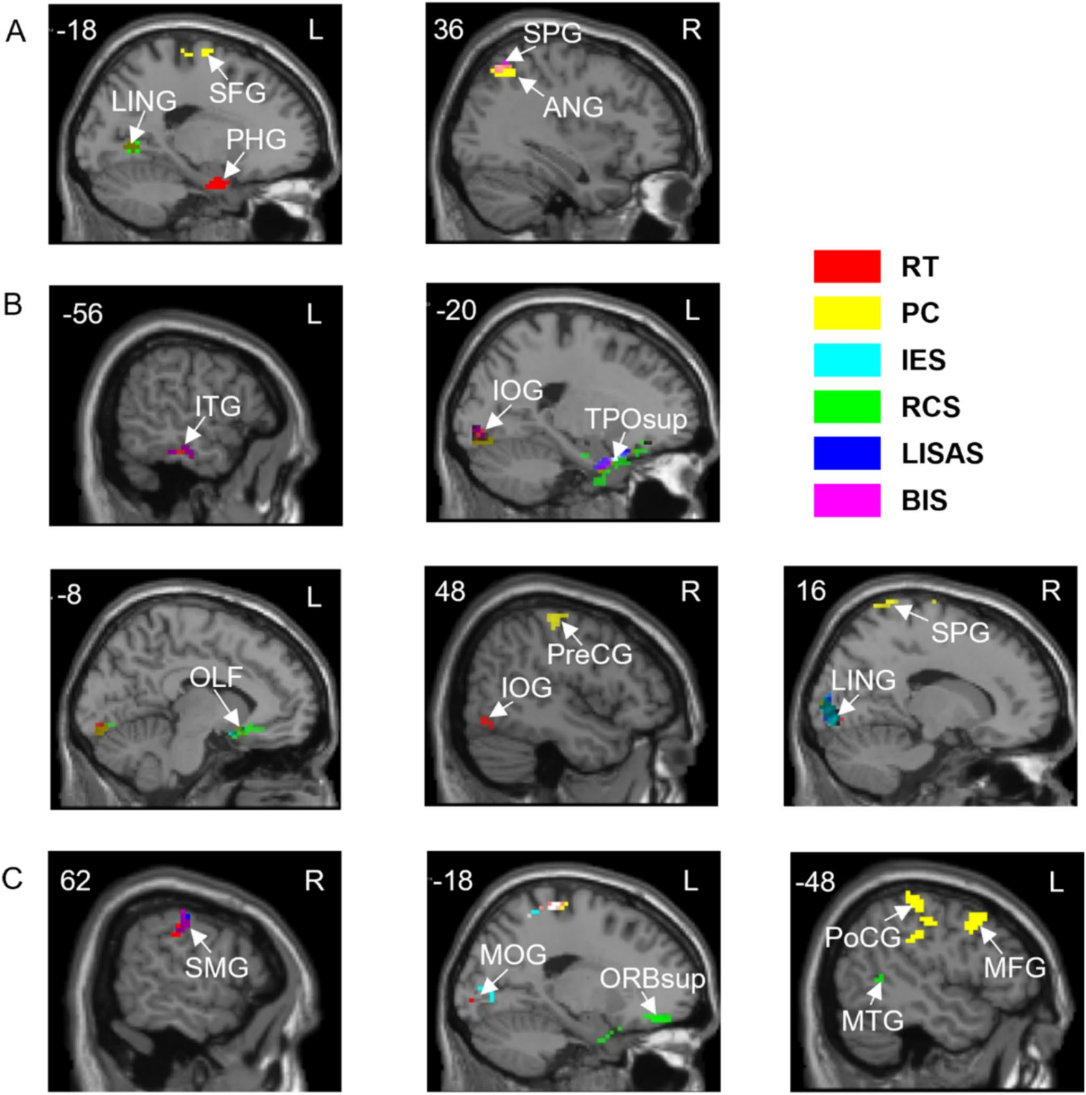
Correlations between brain activation and behavioral indicators in three tasks with spatial cues. **(A)** A task. **(B)** V task. **(C)** VA task. SFG, Superior frontal gyrus; LING, Lingual gyrus; PHG, Parahippocampal gyrus; ANG, Angular gyrus; SPG, Superior parietal gyrus; ITG, Inferior temporal gyrus; IOG, Inferior occipital gyrus; TPOsup, Temporal pole: superior temporal gyrus; OLF, Olfactory cortex; PreCG, Precentral gyrus; SMG, Supramarginal gyrus; MOG, Middle occipital gyrus; ORBsup, Superior frontal gyrus, orbital part; PoCG, Postcentral gyrus; MTG, Middle temporal gyrus; MFG, Middle frontal gyrus. L, left hemisphere; R, right hemisphere. RT, Reaction Time; PC, Proportions of Correct Responses; IES, Inverse Efficiency Score; RCS, Rate-Correct Score; LISAS, Linear Integrated Speed-Accuracy Score; BIS, Balanced Integration Score.

In order to investigate the correlation between task activation and behavioral indicators, Pearson correlation analyses were performed between the activation *β*-values of the three tasks, A, V and VA, and 6 behavioral indicators from spatial, temporal and neutral cues, respectively. The results are shown in Table 2. The brain regions of the A, V and VA tasks are shown in Figure 4.

**Table 2:** Correlations between brain activation and behavioral indicators in three tasks with spatial cues.

Task	Indicator	Brain region	Hemisphere	Coordinates (x, y, z)	*r*	Cluster size
AS	RT	Parahippocampal gyrus	L	−18 0 –27	0.50	38
		Lingual gyrus	L	−18 –63 0	0.41	42
	PC	Angular gyrus	R	33 –66 51	0.48	100
		Superior frontal gyrus	L	−21 –9 72	0.46	57
	RCS	Lingual gyrus	L	−18 –63 0	−0.45	55
	BIS	Superior parietal gyrus	R	30 –66 54	0.44	52
VS	RT	Temporal pole: middle temporal gyrus	L	−24 9 –30	0.57	126
		Inferior temporal gyrus	L	−57 –30 –21	0.47	52
		Inferior occipital gyrus	L	−24 −84 –12	0.48	93
		Lingual gyrus	R	15 –90 –3	0.54	178
	PC	Precentral gyrus	R	33 –21 72	0.55	203
		Superior parietal gyrus	R	24 –33 81	0.58	46
	IES	Temporal pole: superior temporal gyrus	L	−24 6 –30	0.59	80
		Lingual gyrus	R	15 –90 −3	0.50	99
	RCS	Olfactory cortex	L	–3 12 –12	−0.60	243
		Inferior occipital gyrus	L	−24 –84 –12	−0.45	81
		Lingual gyrus	R	15 –90 –3	−0.49	117
	LISAS	Temporal pole: superior temporal gyrus	L	−24 6 –30	0.58	90
		Inferior temporal gyrus	L	−57 –30 –21	0.47	41
		Inferior occipital gyrus	L	−24 –84 –12	0.43	43
		Lingual gyrus	R	15 –90 –3	0.51	110
	BIS	Temporal pole: superior temporal gyrus	L	−24 6 –30	−0.56	41
VAS	RT	Middle occipital gyrus	L	−15 –90 –3	0.46	38
		Supramarginal gyrus	R	63 –21 42	0.41	47
	PC	Postcentral gyrus	L	−27 –42 69	−0.52	444
		Middle frontal gyrus	L	−48 12 45	−0.47	41
	IES	Postcentral gyrus	L	−24 –39 69	0.42	39
	RCS	Inferior frontal gyrus, orbital part	L	−21 15 –24	−0.49	50
		Superior frontal gyrus, orbital part	L	−3 36 –12	0.50	131
		Superior frontal gyrus, orbital part	R	15 48 –18	0.47	45
		Middle temporal gyrus	L	−57 –39 6	0.43	46
	LISAS	Supramarginal gyrus	R	54 –24 36	0.42	47
	BIS	Postcentral gyrus	L	−24 –39 69	−0.49	88

L, left hemisphere; R, right hemisphere.

**Figure 5:**
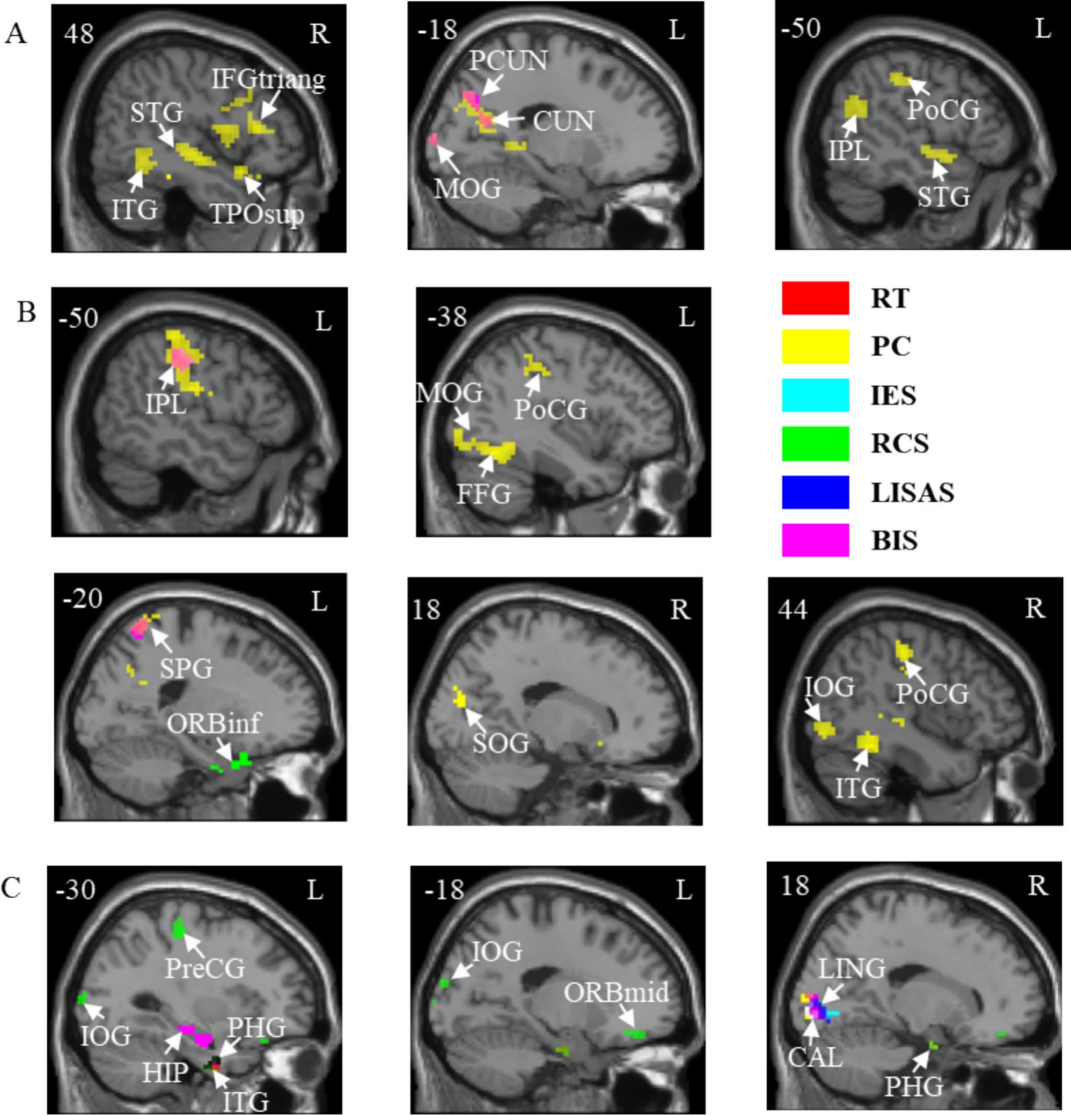
Correlations between brain activation and behavioral indicators in three tasks with temporal cues. **(A)** A task. **(B)** V task. **(C)** VA task. STG, Superior temporal gyrus; ITG, Inferior temporal gyrus; TPOsup, Temporal pole: superior temporal gyrus; IFGtriang, Inferior frontal gyrus, triangular part; PCUN, Precuneus; CUN, Cuneus; MOG, Middle occipital gyrus; IPL, Inferior parietal lobule; PoCG, Postcentral gyrus; FFG, Fusiform gyrus; SPG, Superior parietal gyrus; ORBinf, Inferior frontal gyrus, orbital part; SOG, Superior occipital gyrus; IOG, Inferior occipital gyrus; HIP, Hippocampus; PHG, Parahippocampal gyrus; PreCG, Precentral gyrus; ORBmid, Middle frontal gyrus, orbital part; LING, Lingual gyrus; CAL, Calcarine fissure and surrounding cortex. L, left hemisphere; R, right hemisphere. RT, Reaction Time; PC, Proportions of Correct Responses; IES, Inverse Efficiency Score; RCS, Rate-Correct Score; LISAS, Linear Integrated Speed-Accuracy Score; BIS, Balanced Integration Score.

The results of the correlation between brain activation and behavioral indices for the three tasks under the temporal cues are shown in Table 3. The brain regions for the A, V, and VA tasks are shown in Figure 5.

**Table 3:** Correlations between brain activation and behavioral indicators in three tasks with temporal cues.

Task	Indicator	Brain region	Hemisphere	Coordinates (x, y, z)	*r*	Cluster size
AT	PC	Inferior temporal gyrus	R	60 –36 –24	−0.58	526
		Temporal pole: superior temporal gyrus	R	42 21 –21	0.63	65
		Precuneus	L	−15 –45 3	0.48	87
		Superior temporal gyrus	R	51 –9 –9	0.55	114
		Superior temporal gyrus	L	−51 3 –9	0.43	47
		Inferior frontal gyrus, triangular part	R	42 30 9	0.57	377
		Middle occipital gyrus	L	−36 –93 0	0.45	53
		Cuneus	L	−18 –63 21	0.55	174
		Inferior parietal lobule	L	−33 –78 39	0.52	235
		Postcentral gyrus	L	−48 –21 39	0.44	98
	BIS	Middle occipital gyrus	L	−33 –93 9	0.41	40
		Cuneus	L	−12 –75 36	0.46	84
VT	RT	Parahippocampal gyrus	L	−15 –33 –12	0.52	39
	PC	Fusiform gyrus	L	−42 –54 –15	0.50	187
		Inferior temporal gyrus	R	48 –48 –15	0.51	95
		Inferior occipital gyrus	R	39 –81 –3	0.46	101
		Superior occipital gyrus	R	18 –81 18	−0.48	69
		Postcentral gyrus	L	−51 −30 54	0.46	306
		Middle occipital gyrus	L	–30 –63 33	0.42	51
		Postcentral gyrus	R	48 –21 48	0.46	111
		Superior parietal gyrus	L	−21 –54 72	0.47	59
	RCS	Inferior frontal gyrus, orbital part	L	−21 15 –24	−0.54	55
	BIS	Inferior parietal lobule	L	−51 –30 42	0.40	42
		Superior parietal gyrus	L	−21 –60 63	0.42	51
VAT	RT	Parahippocampal gyrus	R	24 –6 –33	0.48	52
		Inferior temporal gyrus	L	−36 –3 –36	0.53	81
		Calcarine fissure and surrounding cortex	R	21 –87 0	0.45	64
	PC	Lingual gyrus	R	18 –90 –3	−0.53	71
	IES	Lingual gyrus	R	18 –90 –3	0.57	101
	RCS	Parahippocampal gyrus	L	−21 –15 –30	−0.47	62
		Parahippocampal gyrus	R	24 –6 –33	−0.48	50
		Middle frontal gyrus, orbital part	L	−6 36–12	0.55	108
		Inferior occipital gyrus	L	−54 –72 –6	0.41	42
		Precentral gyrus	L	−30 –27 54	0.48	47
	LISAS	Inferior temporal gyrus	L	−36 –3 –36	0.54	55
		Parahippocampal gyrus	R	24 –6 –33	0.48	41
		Lingual gyrus	R	18 –87 –3	0.46	64
	BIS	Hippocampus	L	−30 –12 –21	−0.47	53
		Lingual gyrus	R	18 –90 –3	−0.57	77

L, left hemisphere; R, right hemisphere.

**Figure 6:**
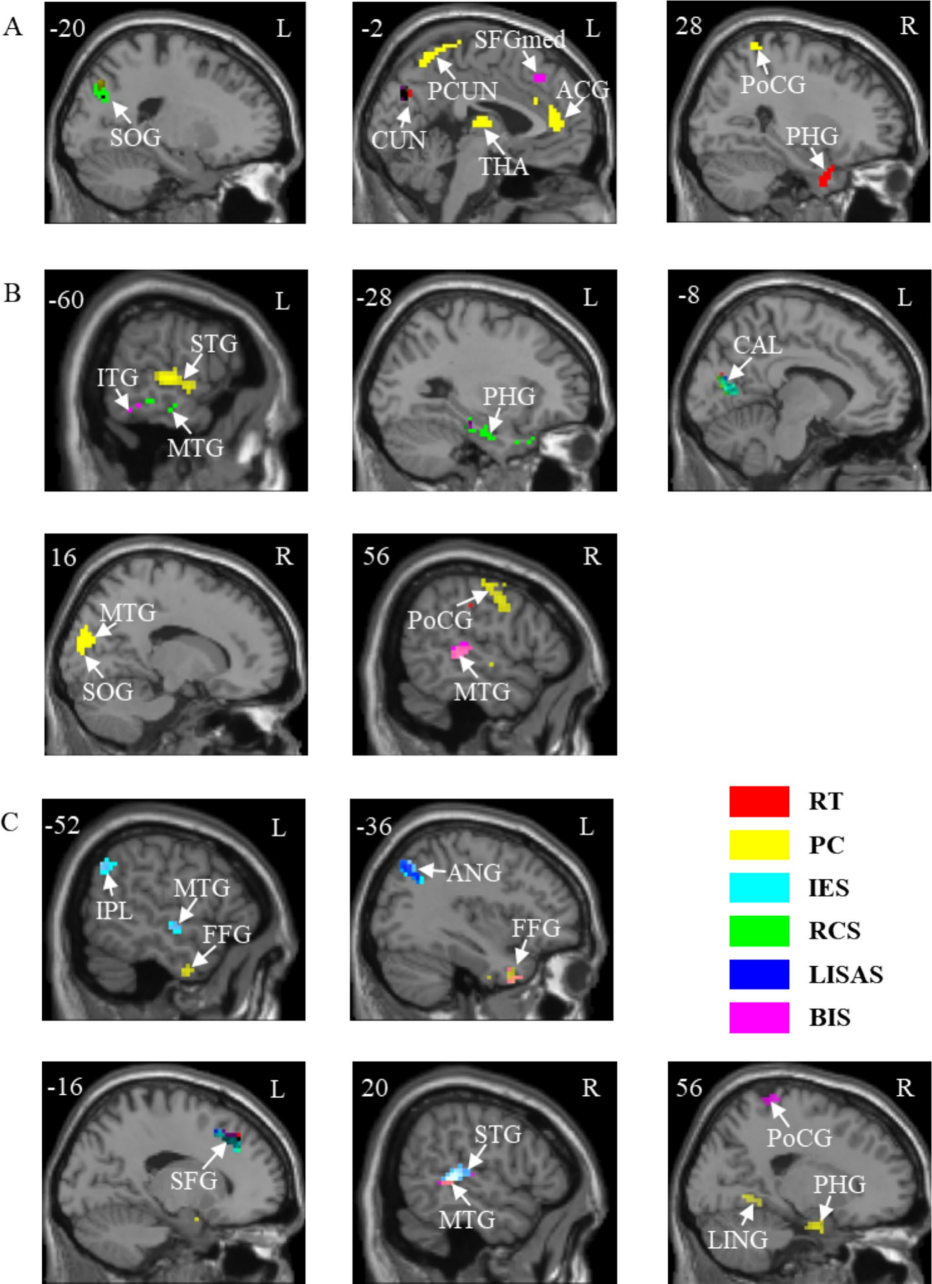
Correlations between brain activation and behavioral indicators in three tasks with neutral cues. **(A)** A task. **(B)** V task. **(C)** VA task. SOG, Superior occipital gyrus; CUN, Cuneus; PCUN, Precuneus; SFGmed, Superior frontal gyrus, medial; ACG, Anterior cingulate and paracingulate gyri; THA, Thalamus; PoCG, Postcentral gyrus; PHG, Parahippocampal gyrus; ITG, Inferior temporal gyrus; MTG, Middle temporal gyrus; STG, Superior temporal gyrus; CAL, Calcarine fissure and surrounding cortex; IPL, Inferior parietal lobule; FFG, Fusiform gyrus; ANG, Angular gyrus; SFG, Superior frontal gyrus; LING, Lingual gyrus. L, left hemisphere; R, right hemisphere. RT, Reaction Time; PC, Proportions of Correct Responses; IES, Inverse Efficiency Score; RCS, Rate-Correct Score; LISAS, Linear Integrated Speed-Accuracy Score; BIS, Balanced Integration Score.

The results of the correlation between brain activation and behavioral indices for the three tasks under neutral cues are shown in Table 4. The brain regions for the A, V, and VA tasks are shown in Figure 6 and Table 5.

**Table 4:** Correlations between brain activation and behavioral indicators in three tasks with neutral cues.

Task	Indicator	Brain region	Hemisphere	Coordinates (x, y, z)	*r*	Cluster size
AN	RT	Parahippocampal gyrus	R	21 –3 –30	0.44	43
		Cuneus	L	−6 –78 30	−0.48	86
	PC	Anterior cingulate and paracingulate gyri	L	−3 42 9	−0.49	108
		Thalamus	L	−15 –21 15	−0.50	148
		Precuneus	L	−6 –39 72	−0.50	58
		Postcentral gyrus	R	36 –45 63	−0.58	86
	RCS	Superior occipital gyrus	L	−18 –75 39	0.45	80
	LISAS	Cuneus	L	−6 –78 30	−0.48	55
	BIS	Superior frontal gyrus, medial	L	−9 39 42	−0.43	45
VN	RT	Parahippocampal gyrus	L	−30 –24 –21	0.41	41
		Calcarine fissure and surrounding cortex	L	−9 –69 9	0.54	69
		Postcentral gyrus	R	66 –12 27	0.48	42
	PC	Middle temporal gyrus	R	60 –33 0	0.40	52
		Superior temporal gyrus	L	−60 –21 3	0.52	164
		Superior occipital gyrus	R	21 –78 18	−0.54	105
		Postcentral gyrus	R	45 –27 57	0.49	209
	IES	Calcarine fissure and surrounding cortex	L	−9 –69 9	0.52	52
	RCS	Parahippocampal gyrus	L	−21 –3 –33	−0.50	154
		Middle temporal gyrus	L	−69 –30 –12	−0.48	40
		Calcarine fissure and surrounding cortex	L	−9 –69 9	−0.52	74
	LISAS	Parahippocampal gyrus	L	−30 –24 –21	0.42	43
		Calcarine fissure and surrounding cortex	L	−9 –69 9	0.52	56
	BIS	Inferior temporal gyrus	L	−45 –15 –24	−0.42	58
		Middle temporal gyrus	R	57 –33 3	0.46	48
VAN	RT	Superior frontal gyrus	L	−15 27 39	−0.59	60
	PC	Fusiform gyrus	L	−27 3 –45	0.69	138
		Parahippocampal gyrus	R	21 –3 –27	0.54	59
		Lingual gyrus	R	27 –54 –3	0.43	48
		Middle temporal gyrus	R	54 –39 0	0.48	53
		Postcentral gyrus	R	45 –27 60	0.53	103
	IES	Middle temporal gyrus	L	−63 –12 –3	−0.46	54
		Superior temporal gyrus	R	57 –30 9	−0.45	57
		Inferior parietal lobule	L	−36 –69 45	−0.51	145
		Superior frontal gyrus	L	−15 27 39	−0.58	44
	RCS	Superior frontal gyrus	L	−15 27 39	0.53	48
	LISAS	Angular gyrus	L	−39 –66 45	−0.43	54
		Superior frontal gyrus	L	−15 27 39	−0.60	58
	BIS	Fusiform gyrus	L	−24 0 –42	0.57	60
		Middle temporal gyrus	L	−66 –15 –3	0.48	43
		Middle temporal gyrus	R	54 –36 3	0.48	78
		Inferior parietal lobule	L	−36 –69 45	0.50	96
		Postcentral gyrus	R	18 –36 72	0.48	47

L, left hemisphere; R, right hemisphere.

**Table 5:** Summary of significant correlation analyses for three tasks with three attentional cues.

Brain	Gyrus	Left hemisphere						Right hemisphere					
		RT	PC	IES	RCS	LISAS	BIS	RT	PC	IES	RCS	LISAS	BIS
Dorsal pathway	Superior frontal gyrus	VAN−	AS +	VAN−	VAS + ^1^ VAN−	VAN−	AN – ^2^				VAS+ ^1^		
	Precentral gyrus				VAT +				VS +				
	Middle frontal gyrus		VAS−		VAT +^2^								
	Superior parietal gyrus		VT +				VT +		VS +				AS +
	Postcentral gyrus		VAS – AT + VT +	VAS +			VAS – VAN +	VN +	VT + AN – VN + VAN +				
	Precuneus		AT + AN−										
	Angular gyrus					VAN−			AS +				
	Supramarginal gyrus							VAS +					
	Superior occipital gyrus				AN +				VT – VN−				
	Cuneus	AN−	AT +			AN−	AT +						
Ventral pathway	Inferior frontal gyrus				VAS−^2^ VT−^2^				AT +^3^				
	Anterior cingulate and paracingulate gyri				VT−								
	Inferior parietal lobule		AT +	VAN−			VT + VAN +						
	Superior temporal gyrus		AT + VN +						AT +	VAN−			
	Middle temporal gyrus			VAN−	VAS + VN−		VAN +		VN + VAN +				VN + VAN +
	Inferior temporal gyrus	VS + VAT +				VS + VAT +	VN−		AT – VT +				
	Temporal pole: superior temporal gyrus			VS +		VS +	VS−		AT +				
	Temporal pole: middle temporal gyrus	VS +											
	Fusiform gyrus		VT + VAN +				VAN +						
	Hippocampus						VAT−						
	Parahippocampal gyrus	AS + VT + VN +			VAT – VN	VN +		VAT + AN +	VAN +		VAT−	VAT +	
	Olfactory cortex				VS−								
	Middle occipital gyrus	VAS +	AT + VT +				AT +						
	Inferior occipital gyrus	VS +			VS – VAT +	VS +			VT +				
	Calcarine fissure and surrounding cortex	VN +		VN +		VN +		VAT +					
	Lingual gyrus	AS +						VS +	VAN +	VS + VAT +		VS + VAT +	
	Thalamus		AN−										

+ positive correlation; − negative correlation.^1^ orbital part; ^2^ medial part; ^3^ triangular part.

## 4 Discussion

In our study, we examined the relationship between brain activation and behavioral representations during audiovisual integration under endogenous attention, specifically focusing on how different types of endogenous attention—spatial, temporal, and neutral—affect behavioral strategies and modulate brain activity along the dorsal and ventral attention pathways. The auditory, visual, and audiovisual tasks based on the classical Posner experimental paradigm were designed. Behavioral data and task-state fMRI data were analyzed to explore differences in activation levels across subjects for the A, V, and VA tasks under different attention conditions. Additionally, we examined whether there were variations in brain region activation, particularly within the dorsal and ventral attention pathways, and explored correlations between activation levels and behavioral representations in each task. This approach highlights the focus on how endogenous attention modulates behavior and neural activity, emphasizing the functional roles of the dorsal and ventral attention pathways in audiovisual integration.

The Visual spatial cue (an arrow), first developed by Posner (1980), providing a spatial hint of the location of an upcoming target stimulus (Ristic and Kingstone, 2006), which are widely used to study the top-down spatial attention. We used 20% ineffective spatial cues to increase visual sensitivity (Solomon, 2004) to enhance attention-directing effects. We used circles as visual temporal cues that provide short or long-time intervals between the cue and the target stimulus, which would guide top-down time-related neural correlations. A study (Olk, 2014) showed that at around 300 ms, the study indicated the presence of a strong cueing effect, whereas greater than 1,500 ms, the cueing effect declined and a clear pattern of inhibition of return emerged. Thus temporal contextual differences in our experiments will affect the allocation of attention, revealing the importance of time-related implicit factors in attention (Olson and Chun, 2001). Neutral cues, on the other hand, are used as nonspecific processes to reveal purely attentional processes. The study demonstrated that we found extensive activation of FEF and IPL in brain activations for all three types of tasks, confirming that the dorsolateral frontoparietal (dFPN) network regulates top-down attention (Ptak et al., 2017) in visual and auditory targets with similarity in neural activation (Smith et al., 2010), suggesting that our experimental design successfully guided the attentional processes. We conducted a practice/training course with all subjects prior to the MR scan, which was conducted to ensure that subjects were familiar with the details of the tasks, and the correctness rate for all three tasks was above 95%, suggesting that the complexity and difficulty of the tasks were balanced. According to the analysis of the behavioral data on the process of audiovisual integration under endogenous attention, the results of the pairwise comparisons of the A, V, and VA tasks showed that there were significant differences in the psychological behavioral indicators including RT, IES, RCS, LISAS, and BIS, and that there was a significant difference in VA versus A and VA versus V for PC. Expressed in the VA task subjects responded more slowly and less correctly, expended more energy on average, responded less efficiently correctly, were less able to switch tasks, and were less able to make judgements about the difficulty of the task. Overall behavioral performance on the VA task was weaker. The classical MLE model assumes that visual and auditory signals of the same origin are integrated into a unified representation and weighted for sensory reliability, but sensory weights are subject to top-down modulation (Vercillo and Gori, 2015). The VA task is more complex than the single-channel tasks, as the brain must attend to information from both the visual and auditory channels, and then make a judgment about whether the stimuli are from the same side before issuing a response. This process is more complex and time-consuming, with sensory weights adjusted due to the spatial inconsistency that may arise in the VA task (Ernst and Banks, 2002; Rohe and Noppeney, 2018). This selective control reduces the gains from multiple senses, and being stimulated on different sides creates challenges in allocating attentional resources (Vatakis et al., 2007). Our findings highlight how the VA task requires different behavioral strategies, particularly in balancing speed and accuracy. The lower values in composite behavioral indicators like LISAS and BIS suggest that the increased complexity of integrating auditory and visual information leads to greater cognitive load, requiring more attention. The differences in these behavioral indicators emphasize the challenge of managing the trade-off between speed and accuracy in the VA task.

Differences in activated brain regions for the three tasks A, V, and VA under spatial, temporal, and neutral cues showed consistency in neural-level characterization, including the frontal eye field, inferior parietal lobule., supplementary motor area, superior temporal gyrus, middle occipital gyrus and cuneus brain regions. The frontal eye field, inferior parietal lobule and supplementary motor area belong to the dorsal frontoparietal network, an endogenous attentional task-activated brain region (Tang et al., 2016). These regions are involved in the regulation of spatial attention and task-related processing. The frontal eye field, inferior parietal lobule and superior temporal gyrus belonging to the association cortex, which processes information from visual & auditory channels and is responsible for audiovisual semantic integration (Li et al., 2016). The middle occipital gyrus and cuneus are primarily associated with visual information processing and belong to the primary visual cortex. These regions’ activation patterns highlight how attention networks, particularly the dorsal pathway, help allocate cognitive resources during task processing and facilitate the integration of audiovisual information.

In a correlation analysis of activation levels with behavioral indicators (Table 5), we used two basic behavioral metrics and four composite metrics and found that brain activity in multiple brain regions was behaviorally relevant and overlapped with task-related activation in three tasks, A, V, and VA, under spatial, temporal, and neutral cues. These overlapping brain regions suggest a direct link between their activity patterns during task performance and behavioral performance. The cuneus, lingual gyrus, and middle occipital gyrus are associated with visual information processing, and the inferior temporal gyrus and middle temporal gyrus are associated with visual information processing and belong to more advanced regions of visual processing. The angular gyrus and supramarginal gyrus are engaged in auditory information processing, with the angular gyrus also playing a role in decision-making by directing attention to relevant reward-related information in the visual environment (Studer et al., 2014). The fusiform gyrus, part of the multimodal association cortex, integrates sensory inputs from both visual and auditory channels. The frontal eye field, inferior parietal lobule, and superior parietal gyrus belong to the dorsal frontoparietal network and are involved in spatial attention and task-related focus (Chica et al., 2013), playing a crucial role in directing attentional resources. We found correlations with PC and BIS in the overlapping left Inferior parietal lobule, suggesting that IPL relies on accuracy in auditory tasks guided by visual endogenous attention, while top-down integration is enhanced in visual or audiovisual to balance overall task performance. The ventral attention network, including regions like the angular gyrus and superior temporal gyrus, is more stimulus-driven, helping the brain shift attention to relevant stimuli. We found in the overlapping bilateral Superior temporal gyrus that participation in endogenous attentional guidance in a single-channel task improves task completion by modulating information processing in a single modality to accomplish bottom-up allocation of attention. In addition, the hippocampus, parahippocampal gyrus, and temporal pole are associated with memory storage (Wagner et al., 2020; Setton et al., 2022), but also contribute to spatial perceptual learning and recognition of landmark objects (Jeffery, 2018; Sun et al., 2021), reflecting broader attentional modulation. These findings highlight how both dorsal and ventral attention pathways are engaged in managing cognitive load and balancing the integration of visual and auditory information.

In RT and PC correlations with brain activation we found broader significance results, with PC being more broadly significant than RT, suggesting that better PCs (higher grades) require more mobilization of perceptual resources than better RTs (faster speeds) (Helfrich et al., 2019; Hunter, 2021). In terms of RT (faster), we only observed a negative correlation of brain region activation accompanied by RT in neutral attention, which does suggest that increased information processing leads to slower reaction times when more brain activation intervenes, which may be related to the deliberate decision-making performed by the subjects (average task PC > 95%) (Vallesi et al., 2021; Schneider et al., 2022). Unlike single-channel stimuli, we observed differences in the correlation between RT and PC for spatial & temporal attention in the VA task, where increased brain activated areas resulted in increased RT and decreased PC, which is the opposite of neutral attention, and is manifested in areas dominated by sensory integration and visuo-spatial processing, suggesting that increased top-down spatio-temporal modulation mobilizes additional cognitive resources and thus affects the task performance strategy (Vidaud-Laperrière et al., 2022; Carmona et al., 2024). Improvements in task performance require greater mobilization of perceptual resources, while increased task complexity under different attentional modalities may alter an individual’s execution strategy, thereby affecting the relationship between reaction time and performance scores. Spatial and temporal attention are modulated, and individuals not only mobilize more cognitive resources, but may also respond to the challenges of the task by adjusting their task strategies.

In the IES we did not obtain a significance correlation in the A task, and in the V task we obtained an increase in spatial and neutral attention for the average energy consumed by the system over trials (Townsend, 1983) in the visual processing area. This exacerbates the energetic deepening of visual space in spatial attention, which differs from the monitoring function of visual targets in neutral attention, and follows the same trend as in VA, as evidenced by increased activation of visually and spatially relevant brain regions consuming energy in spatial and temporal attention, and energy savings in higher cognitive regions in neutral attention. In correlation with the RCS then characterized the efficiency of correct response trials, both of which negatively correlated with brain activation in the V task, as demonstrated by the processing of basic visual sensory information in spatial attention and the processing of higher cognition in temporal & neutral attention. This indicates that under spatial attention, the processing of basic visual information is associated with higher cognitive processing under temporal and neutral attention, suggesting that there are differences in top-down attentional modulation of cognitive strategies (Hahn et al., 2006; Li et al., 2012). Both LISAS and BIS are balanced for speed-accuracy, and the correlation differences between these two sets of metrics reveal differences between linear and nonlinear processing patterns in balancing cognitive resources in different brain regions. Our results reveal that the introduction of spatio-temporal attentional modulation in the A task increases visuo-spatial orienting processing would show accuracy, whereas in neutral attention it affects behavioral performance. This is the same in both indicators, whereas the LISAS and BIS show differential processing patterns after the addition of visual stimuli (V&VA task) and are completely reversed in the VA task, suggesting a dynamic modulation of the top-down attentional orientated processing audiovisual integration (Todd and Manaligod, 2018; Zeraati et al., 2023). Different attentional modalities not only modulate the brain’s workload, but also influence the efficiency of task performance, especially the balance between visuo-spatial and higher cognitive processing. Furthermore, audiovisual integration performance was affected by the modulation of top-down attention, suggesting that the brain optimizes task speed and accuracy through flexible resource allocation in the face of multimodal information.

## 5 Conclusion

Three audiovisual tasks based on the Posner experimental paradigm were designed to calculate behavioral indicators, brain region activation differences, and correlations between brain activation and behavioral representations under spatial, temporal, and neutral cues, respectively, for each task. Significant differences in behavioral performance were found across tasks, with the dual-channel task performing weaker than the single-channel task. Consistent and widespread brain activation was demonstrated in the frontal eye field, inferior parietal lobule, supplementary motor area, superior temporal gyrus, middle occipital gyrus and cuneus brain regions. In the correlations between behavioral indicators and brain activation, PC had broader differential results. In audiovisual tasks, spatio-temporal attentional modulation mobilizes additional cognitive resources and influences executive strategies compared to neutral attention. IES reveals endogenous attentional modulation of mean energy expenditure in dual-channel tasks, and the RCS reveals between-task differences in the efficiency of responding correctly. LISAS and BIS show different patterns of speed-accuracy balance in audiovisual tasks, indicating dynamic modulation of cognitive processes by attention networks. Dual attention pathways are involved in managing cognitive load, balancing the integration of visual and auditory information, and influencing task performance strategies.

## Data Availability

The original contributions presented in the study are included in the article/supplementary material, further inquiries can be directed to the corresponding authors.

## Footnotes

^1^ http://www.neurobs.com/

^2^ https://www.fil.ion.ucl.ac.uk/spm

^3^ http://www.restfmri.net/forum/REST
