# Correction: Behavioral representations within the endogenous dual attentional pathways during audiovisual integration processing

**DOI:** 10.3389/fnins.2025.1738133

**Published:** 2025-11-18

**Authors:** Zhixi Zhang, Zhongtian Guan, Miao He, Yubo Liu, Mingli Yan, Chunlin Li

**Affiliations:** 1 School of Biomedical Engineering, Capital Medical University, Beijing, China; 2 Aerospace Information Research Institute, Chinese Academy of Sciences, Beijing, China; 3 Institute of Large-Scale Scientific Facility, Beihang University, Hangzhou, Zhejiang, China

**Keywords:** fMRI, endogenous attention pathway, audiovisual integration, Posner cueing paradigm, behavioral representations

Affiliation “School of Biomedical Engineering, Capital Medical University, Beijing, China” was omitted for author Zhixi Zhang. This affiliation has now been added for author Zhixi Zhang.

The order of authors in the author list of the published paper was erroneously given as:

Zhongtian Guan^**†**^, Mingli Yan^**†**^, Miao He, Yubo Liu, Zhixi Zhang^*^, Chunlin Li^*^

^**†**^These authors have contributed equally to this work.

The correct author list reads:

Zhixi Zhang^**†**^, Zhongtian Guan^**†**^, Miao He, Yubo Liu, Mingli Yan^*^, Chunlin Li^*^

^**†**^These authors have contributed equally to this work.

Author Zhixi Zhang was erroneously assigned as a corresponding author. The correct corresponding author is Mingli Yan.

Author Mingli Yan was erroneously assigned as equal contributing author.

Author Zhixi Zhang was erroneously omitted as equal contributing author.

The original version of this article has been updated.

