# Correction: Behavioral representations within the endogenous dual attentional pathways during audiovisual integration processing

**DOI:** 10.3389/fnins.2026.1915761

**Published:** 2026-07-08

**Authors:** Zhixi Zhang, Zhongtian Guan, Miao He, Yubo Liu, Mingli Yan, Chunlin Li

**Affiliations:** 1 School of Biomedical Engineering, Capital Medical University, Beijing, China; 2 Aerospace Information Research Institute, Chinese Academy of Sciences, Beijing, China; 3 Institute of Large-Scale Scientific Facility, Beihang University, Hangzhou, Zhejiang, China

**Keywords:** fMRI, endogenous attention pathway, audiovisual integration, Posner cueing paradigm, behavioral representations

In the **Acknowledgments**, information was erroneously omitted. The updated **Acknowledgments** Section is as follows:

We express our gratitude to all those who provided assistance and thank every participant for their active cooperation. We also thank Dr. Liwei Sun for his assistance with the experimental paradigm, including advice on paradigm logic, stimulus materials, and audiovisual stimulus presentation.

The funder [Capital Medical University Clinical-Basic Research Collaborative Platform Development Project], [JLPYPT2025007] was erroneously omitted.

The original version of this article has been updated.

